# Bioinformatics Analysis Reveals an Association Between Cancer Cell Stemness, Gene Mutations, and the Immune Microenvironment in Stomach Adenocarcinoma

**DOI:** 10.3389/fgene.2020.595477

**Published:** 2020-12-11

**Authors:** Zaisheng Ye, Miao Zheng, Yi Zeng, Shenghong Wei, Yi Wang, Zhitao Lin, Chen Shu, Yunqing Xie, Qiuhong Zheng, Luchuan Chen

**Affiliations:** ^1^Department of Gastrointestinal Surgical Oncology, Fujian Cancer Hospital and Fujian Medical University Cancer Hospital, Fuzhou, China; ^2^Department of Clinical Laboratory, Fujian Maternity and Child Health Hospital, Affiliated Hospital of Fujian Medical University, Fuzhou, China; ^3^Department of Fujian Provincial Key Laboratory of Tumor Biotherapy, Fujian Cancer Hospital and Fujian Medical University Cancer Hospital, Fuzhou, China

**Keywords:** stomach adenocarcinoma, cancer stemness, clinical characteristics, tumor microenvironment, tumor mutation burden

## Abstract

Cancer stem cells (CSCs), characterized by infinite proliferation and self-renewal, greatly challenge tumor therapy. Research into their plasticity, dynamic instability, and immune microenvironment interactions may help overcome this obstacle. Data on the stemness indices (mRNAsi), gene mutations, copy number variations (CNV), tumor mutation burden (TMB), and corresponding clinical characteristics were obtained from The Cancer Genome Atlas (TCGA) and UCSC Xena Browser. The infiltrating immune cells in stomach adenocarcinoma (STAD) tissues were predicted using the CIBERSORT method. Differentially expressed genes (DEGs) between the normal and tumor tissues were used to construct prognostic models with weighted gene co-expression network analysis (WGCNA) and Lasso regression. The association between cancer stemness, gene mutations, and immune responses was evaluated in STAD. A total of 6,739 DEGs were identified between the normal and tumor tissues. DEGs in the brown (containing 19 genes) and blue (containing 209 genes) co-expression modules were used to perform survival analysis based on Cox regression. A nine-gene signature prognostic model (ARHGEF38-IT1, CCDC15, CPZ, DNASE1L2, NUDT10, PASK, PLCL1, PRR5-ARHGAP8, and SYCE2) was constructed from 178 survival-related DEGs that were significantly related to overall survival, clinical characteristics, tumor microenvironment immune cells, TMB, and cancer-related pathways in STAD. Gene correlation was significant across the prognostic model, CNVs, and drug sensitivity. Our findings provide a prognostic model and highlight potential mechanisms and associated factors (immune microenvironment and mutation status) useful for targeting CSCs.

## Introduction

Globally, stomach cancer is the fifth leading cancer (7% of all cases) and the third leading cause of cancer-related death, accounting for 9% of deaths (Brenner et al., 2009). The survival and prognosis of stomach cancer remain poor, despite significant advances in treatment options, such as surgery, chemotherapy, radiation therapy, immunotherapy, and gene therapy (Tan, 2019). Most patients with stomach cancer are diagnosed with metastasis, commonly localized to the liver, abdominal lining, lungs, lymph nodes, and bones (Digklia and Wagner, 2016). Many factors are crucial for the development of gastric cancer, including *Helicobacter pylori* infection, smoking, alcohol, and smoked food consumption, obesity, genetic syndromes, and chronic atrophic gastritis (Poorolajal et al., 2020). Delineating the complicated mechanisms involved in stomach cancer pathogenesis can help mitigate its spread.

Cancer stem cells (CSCs) can self-renew, proliferate infinitely, and form heterogeneous tumor cell populations. The mRNA expression-based stemness index (mRNAsi) was used to quantify stemness. Higher mRNAsi scores are associated with active biological processes in CSCs and greater tumor dedifferentiation, as reflected by histopathological grades (Vlashi and Pajonk, 2015). Compared with cancer cells without stemness, CSCs possess enhanced ability for tumor progression, drug resistance, metastasis, and self-renewal through self-protection mechanisms, such as inhibition of apoptosis pathways, DNA damage repair, and production of drug-resistant proteins (Beck and Blanpain, 2013). To solve the difficulties associated with cancer treatment, researchers are searching for drugs that specifically target CSC surface markers or related signaling pathways (Dawood et al., 2014). Recently, an increasing number of studies have reported on the factors that affect the dynamic instability of CSCs, such as gene heterogeneity and the mutual influence of the tumor immune microenvironment (Miranda et al., 2019). For example, the tumor immune microenvironment of CSCs consistently infiltrates several natural killer cells (NKs), cytotoxic CD8^+^ T cells (CD8^+^ T), CD4^+^ T helper cells (CD4^+^ T), tumor-associated macrophages, and tumor-associated neutrophils (Ahmed et al., 2018). These immune cells in the CSC niche play an important role in enabling the evasion of immune surveillance and induce tumor growth, migration, and stemness maintenance (Vahidian et al., 2019). Additional epigenetic and mutational events also induce CSC emergence and adenocarcinoma, including stomach adenocarcinoma (STAD) (Bessède et al., 2015). Some stemness factors such as Sox2, Oct3, Oct4, and Nanog are related to pluripotent stem cells in STAD (Akhavan-Niaki and Samadani, 2014). The stemness of STAD was also similarly dependent on cancer-related signaling pathways, such as Notch and mTORC1 signaling, to promote gastric cancer cell proliferation. These studies demonstrated that targeting Notch and mTOR pathways in combination might be a potential therapeutic strategy for patients with STAD (Hibdon et al., 2019). Research progress on the targeting effect and regulation of CSCs has focused on cytokines, signal transduction pathway inhibitors, non-coding RNAs, and other CSC-related proteins (Gulaia et al., 2018). Moreover, the effective and persistent antitumor activity of HER2-directed chimeric antigen receptor T cells affected the maintenance of *in vitro* CSC subpopulations of STAD and xenotransplanted *in vivo* tumors. The development and clinical application of adoptive immunotherapy targeting CSCs in patients with STAD may be possible in the near future (Song et al., 2018).

Here, we identified and explored CSC-related differentially expressed genes (DEGs) in STAD, the related biological processes (BP), and enhanced pathways to better understand their functions. Furthermore, the prognostic model based on the stemness index in STAD showed strong relevance to the clinical characteristics and survival rates. All genes in the prognostic model were crucial; some were divided by copy number variations (CNV), and some were divided by gene mutations. The correlation between gene expression and drug sensitivity is important for proper guidance of CSC drug research in the future. The whole prognostic model was evaluated using a risk score. We obtained insights into the interface of the infiltrating immune system cells, tumor mutation burden (TMB), and pathways related to stemness in STAD.

## Materials and Methods

### Genome-Wide Omics Data in STAD

The gene expression RNAseq (HTSeq-FPKM GDC Hub) and corresponding clinical characteristics were downloaded from The Cancer Genome Atlas (TCGA) website^1^. The clinical characteristics included sex (male and female), age (from 35 to 90 years old), pathologic T (tumor size, including T1, T2, T3, T4, and TX), pathologic M (tumor metastasis, including M0, M1, and MX), pathologic N (tumor lymph node metastasis, including N0, N1, N2, and NX), pathologic stage (stages I, II, III, and IV), and survival information (survival time and status). The data for mRNA expression-based stemness index (mRNAsi) (stemness score-RNA based on Pan-Cancer Atlas Hub), somatic mutation (VarScan2 Variant Aggregation and Masking), and CNV [GISTIC-focal score by gene Genomic Data Commons (GDC) Hub] were downloaded from UCSC Xena datasets^2^. The Maftools R package^3^ was used to calculate the distribution of TMB according to somatic mutation data, which also generated the waterfall plot of mutation genes.

The stemness of STAD cancer cells was evaluated using mRNAsi (0–1). The closer the value was to 1, the stronger the stemness characteristics of the cancer cells. The Kaplan–Meier survival analysis method was used to describe the overall survival of patients with STAD based on mRNAsi, which divided mRNAsi into two groups (high and low mRNAsi groups). *P* < 0.05 was considered a significant difference.

### Identification of DEGs Between Normal and Tumor Tissues in STAD

The limma package^4^ was used for the analysis of DEGs between normal and tumor tissues in STAD. The inclusion criteria for DECs were *p* < 0.05, false discovery rate (FDR) filter ≤ 0.05, and log (fold change) filter ≥ 1.

### Weighted Gene Co-expression Network Analysis to Select the Co-expression Modules and Hub Genes

The identified DEGs between normal and tumor tissues in STAD were used to construct co-expression modules with the weighted gene co-expression network (WGCNA) R package^5^. The goodSamplesGenes function in the WGCNA R package was used to test the data quality of the included samples (Li and Zhan, 2019). Pearson correlation coefficients between each gene module were used to construct a matrix to establish the module-trait relationship between DEG expression and the corresponding mRNAsi according to the β value (soft-threshold value). The modules with the highest correlation to both mRNAsi and EREG-mRNAsi were selected for further research. Genetic significance (GS) was represented as the level of correlation between DEG expression and mRNAsi. The GS for mRNAsi was also calculated using linear regression. The inclusion criteria for a hub gene were set as follows: module membership > 0.8, and GS > 0.5.

### Cox Regression Survival and Functional Enrichment Analyses of DEGs in Blue and Brown Co-expression Modules

Cox regression analysis was used to calculate the regression coefficient for each hub of DEGs between normal and tumor tissues in blue and brown co-expression modules. We used the survival R package^6^ to obtain survival-related DEGs and construct a prognostic model. Survival-related DEGs were analyzed using the Kyoto Encyclopedia of Genes and Genomes (KEGG) pathways^7^ to explore the cancer-involved signaling pathways. Gene Ontology terms analysis (Cytoscape v3.8) was used to analyze the BP of the survival-related DEGs. *P* < 0.05, and FDR < 0.05 were applied as the inclusion criteria for KEGG pathway and GO analysis. The enrichment *P*-value was calculated based on 10,000 permutations, and the FDR value was calculated with the Benjamini–Hochberg multiple testing correction procedure. The protein and protein interaction (PPI) network was used to construct an interaction network of the survival-related DEGs^8^.

### Lasso Regression Construction and Verification of STAD

Survival-related DEGs (risk ratio > 1.4 or risk ratio < 0.7, *p* < 0.05) were selected to construct Lasso regression using the glmnet R package^9^ (Li and Zhan, 2020). The STAD samples were divided into high- and low-risk score groups according to the median risk score. The multiple receiver operating characteristic (ROC) curve was plotted using the R package^10^ to show the area under the curve (AUC) of risk score, age, sex, grade, stage, T stage, M stage, and N stage in STAD. Principal component analysis (PCA) was used to test whether the risk score was a good measurement for sample classification in STAD. The Kaplan–Meier method evaluated the relevance of overall survival in risk score groups. Corrplot R package^11^ was used to perform the correlation analysis between the expression of DEGs in the prognostic model and CNV using the Spearman method (*p* < 0.05). The association between the expression of DEGs in the prognostic model and drug sensitivity was performed using the Corrplot R package with the Pearman method (*p* < 0.05) based on the corresponding data from CellMiner^12^.

### Proportions of Immune Cells in STAD Based on the CIBERSORT Method

To quantify the proportions of immune cells in STAD, the CIBERSORT algorithm with the LM22 gene signature was used. This is a popular method to discriminate between 22 human immune cell phenotypes (naïve B cells, memory B cells, naïve CD4^+^ T cells, resting memory CD4^+^ T cells, activated CD4^+^ T cells, CD8^+^ T cells, gamma delta T cells, follicular helper T cells, regulatory T cells (Tregs), plasma cells, resting NK cells, activated NK cells, M0 macrophages, M1 macrophages, M2 macrophages, monocytes, resting mast cells, activated mast cells, resting dendritic cells, activated dendritic cells, eosinophils, and neutrophils). Gene expression profiles of STAD from the TCGA database were uploaded to the CIBERSORT web portal^13^ with the algorithm running 1,000 permutations.

### Correlation of Risk Score Based on the Prognostic Model With Clinical Characteristics and Immune Cells

The correlation of clinical characteristics, including age at initial diagnosis, sex, pathologic M, pathologic N, pathologic T, pathologic stage, and cancer status, between the high- and low-risk score groups was performed using the pheatmap R package^14^. Additionally, the clinical characteristics and risk scores of patients with STAD were analyzed using univariate and multivariate Cox regression survival models^15^. The clinical characteristics and risk score-based assessment nomogram^16^ were used to evaluate prognosis in STAD patients (1-, 2-, and 3-year survival rates). Different proportions of immune cells between the high- and low-risk score groups analyzed using the ggpubr R package^17^ (*p* < 0.05). Furthermore, the correlation of each proportion of immune cells between the high- and low-risk score groups was analyzed using the Corrplot R package (*p* < 0.05). The correlation between the risk score and TMB was plotted using GraphPad Prism 8 with Spearman’s software (*p* < 0.05).

### Gene Set Enrichment Analysis Identified Different Gene Sets Between the High- and Low-Risk Score Groups

Gene set enrichment analysis (GSEA) (version 4.1.0) was used to identify classes of genes associated with phenotypes of risk scores that were overrepresented in a large set of genes. The enrichment *P*-value was calculated based on 1,000 permutations, and the FDR value was calculated with the Benjamini-Hochberg multiple testing correction procedure (*p* < 0.05, FDR *q*-val < 0.05).

## Results

### mRNAsi Was Significantly Associated With STAD

**FIGURE 1:**
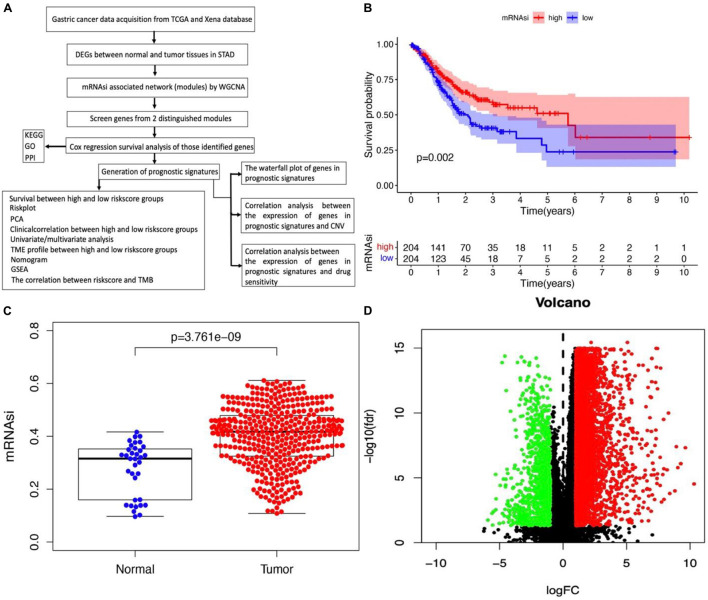
Identification of DEGs betweennormal and tumor tissues in STAD. **(A)** The flow chart for identification of mRNAsi related signature in STAD. **(B)** Kaplan–Meier curves show that the low mRNAsi group had greater mortality than the high mRNAsi group. **(C)** Differences in mRNAsi between normal and tumor tissues in STAD. **(D)** Identification of DEGs between normal and tumor tissues in STAD; green and red indicate downregulated and upregulated genes, respectively.

The flowchart shown in Figure 1A summarizes the overall bioinformatics analysis of the association between cancer stemness, gene mutations, and immune response in STAD. The data of mRNAsi were used to perform the overall survival analysis in STAD (Supplementary Table 1). The mRNAsi subgroups were clustered according to the median value of mRNAsi in STAD. The overall survival was significantly different between the high and low mRNAsi groups (Figure 1B, *p* = 0.002). A significant difference in mRNAsi was observed between normal and tumor tissues in STAD (Figure 1C). A total of 6,739 DEGs were identified between normal and tumor tissues in STAD, of which 1,146 were upregulated and 5,593 were downregulated (Figure 1D and Supplementary Table 2).

### WGCNA: Identification of the Most Significant Modules and Genes

**FIGURE 2:**
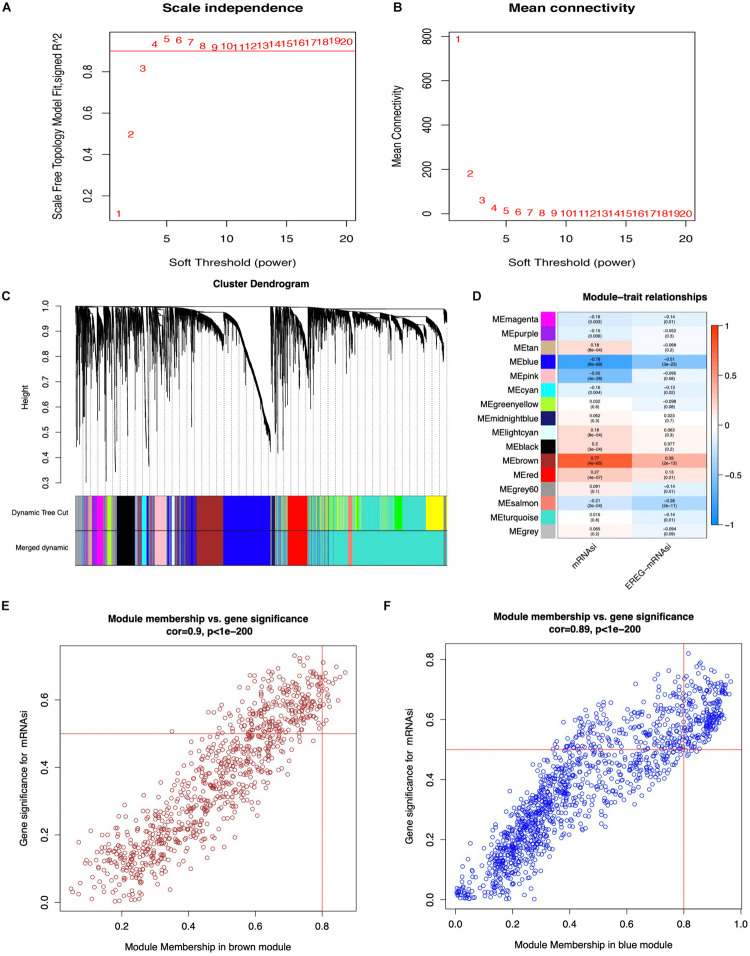
Weighted gene co-expression network of STAD. **(A,B)** Soft threshold to identify the WGCNA module. **(C)** The cluster dendrogram of co-expression modules in STAD. **(D)** Correlation between the gene module and clinical traits, including mRNAsi and EREG-mRNAsi. **(E,F)** Scatter plot of module eigen genes in the blue and brown co-expression modules.

WGCNA was performed to select the most significant gene modules associated with STAD stemness. When β = 4 (a soft threshold), the scale-free *R*^2^ was 0.940 to obtain a higher average connectivity degree (Figures 2A,B). The 6,739 DEGs were clustered into 16 gene co-expression modules, including black, blue, brown, cyan, green-yellow, gray, gray60, light cyan, magenta, midnight blue, pink, purple, red, salmon, tan, and turquoise (Figures 2C,D). In the identified gene co-expression modules, the black and brown co-expression modules were most significantly related to mRNAsi and EREG-mRNAsi. The blue module was significantly associated with mRNAsi, with a correlation close to -0.78, and with EREG-mRNAsi, with a correlation close to -0.51. The brown module was significantly associated with mRNAsi, with a correlation of 0.77, and with EREG-mRNAsi, with a correlation of 0.39. In addition, the correlation between module membership and GS in the brown co-expression module was significantly associated with mRNAsi, with a correlation of 0.9 (Figure 2E, and *p* < 0.05). The correlation between module membership and GS in the blue co-expression module was significantly associated with mRNAsi, with a correlation of 0.89 (Figure 2F, and *p* < 0.05). Thus, blue and brown co-expression modules were used for subsequent analyses. Furthermore, hub genes were identified in the blue and brown co-expression modules, with module membership > 0.8 and GS > 0.5. There were 19 and 209 hub genes in the brown and blue co-expression modules, respectively (Supplementary Table 3).

### Survival Analysis and Functional Enrichment of Hub DEGs in the Brown and Blue Co-expression Modules

**FIGURE 3:**
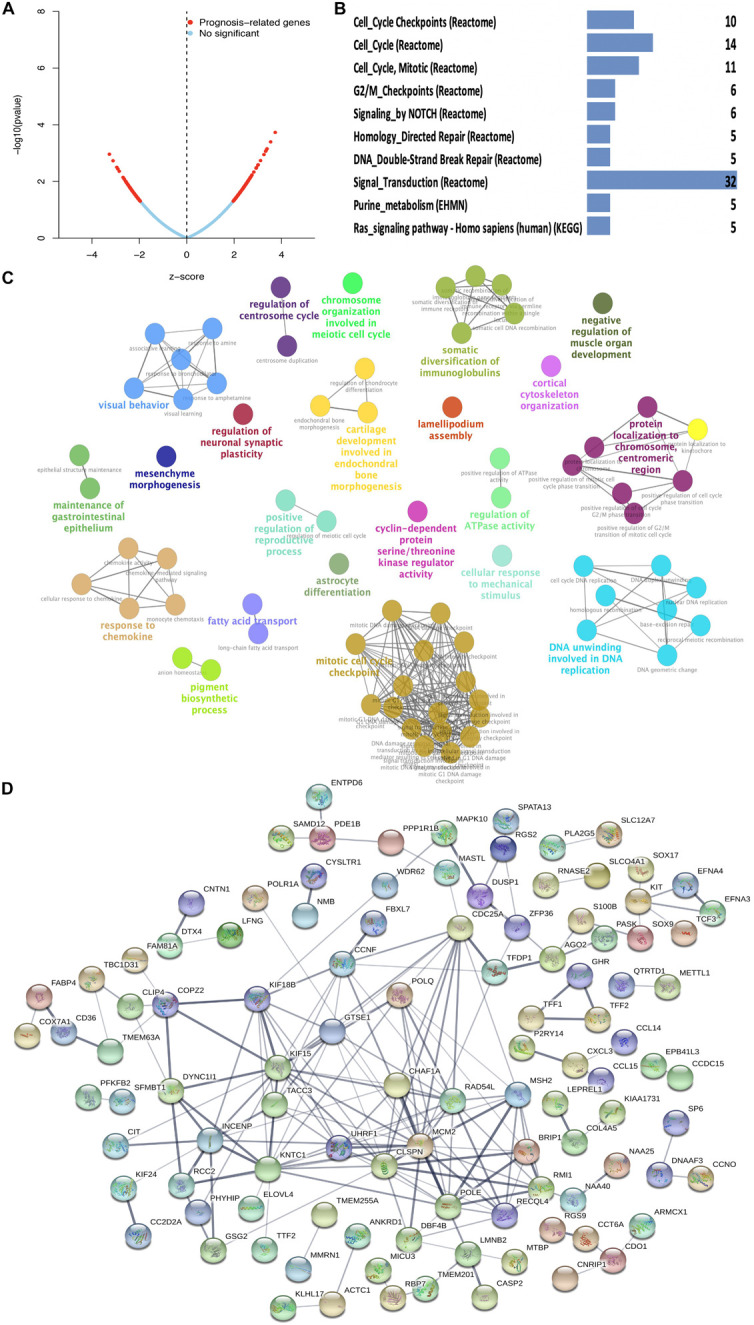
Function enrichment analysis of survival-related DEGs in STAD. **(A)** Prognosis-related genes by Cox regression in blue and brown co-expression modules. **(B)** KEGG pathway of survival-related DEGs in blue and brown co-expression modules. **(C)** GO-BP analysis of survival-related DEGs in blue and brown co-expression modules. A lower *P*-value and more significant enrichment were shown with greater node size. The same color indicated the same function group. Among the groups, we chose a representative of the most significant term and lag highlighted. **(D)** PPI network of survival-related DEGs in blue and brown co-expression modules.

The 228 hub genes identified in the brown and blue co-expression modules were used for survival analysis with Cox regression analysis. A total of 178 hub DEGs in the brown and blue co-expression modules were significantly related to the risk ratio in STAD (Figure 3A and Supplementary Table 4). These survival-related hub DEGs were further enriched for pathway analysis, and 12 significant pathways were obtained, including cell cycle checkpoints, cell cycle, cell cycle (mitotic), G2/M checkpoints, NOTCH signaling, homology directed repair, DNA double-strand break repair, signal transduction, purine metabolism, and Ras signaling pathways (Figure 3B and Supplementary Table 5). These survival-related hub DEGs also showed GO enrichment according to BP to explore the potentially involved mechanisms of action of CSCs. Seventy-two BP enrichments were obtained, including maintenance of gastrointestinal epithelium, mesenchyme morphogenesis, response to chemokines, regulation of neuronal synaptic plasticity, somatic diversification of immunoglobulins, fatty acid transport, mitotic cell cycle checkpoint, regulation of centrosome cycle, cyclin-dependent protein serine/threonine kinase regulator activity, lamellipodium assembly, regulation of ATPase activity, DNA unwinding involved in DNA replication, protein localization to chromosome, centromeric region, and cortical cytoskeleton organization (Figure 3C and Supplementary Table 6). The PPI network obtained a high combined score and co-expression correlation between some of the interaction proteins. For example, UHRF1 and MCM2, CHAF1A and MCM2, MCM2 and MSH2, ZFP36 and DUSP1, POLE and MCM2, and POLE and MSH2 (Figure 3D and Supplementary Table 7).

### Construction of a Prognostic Model for STAD

**FIGURE 4:**
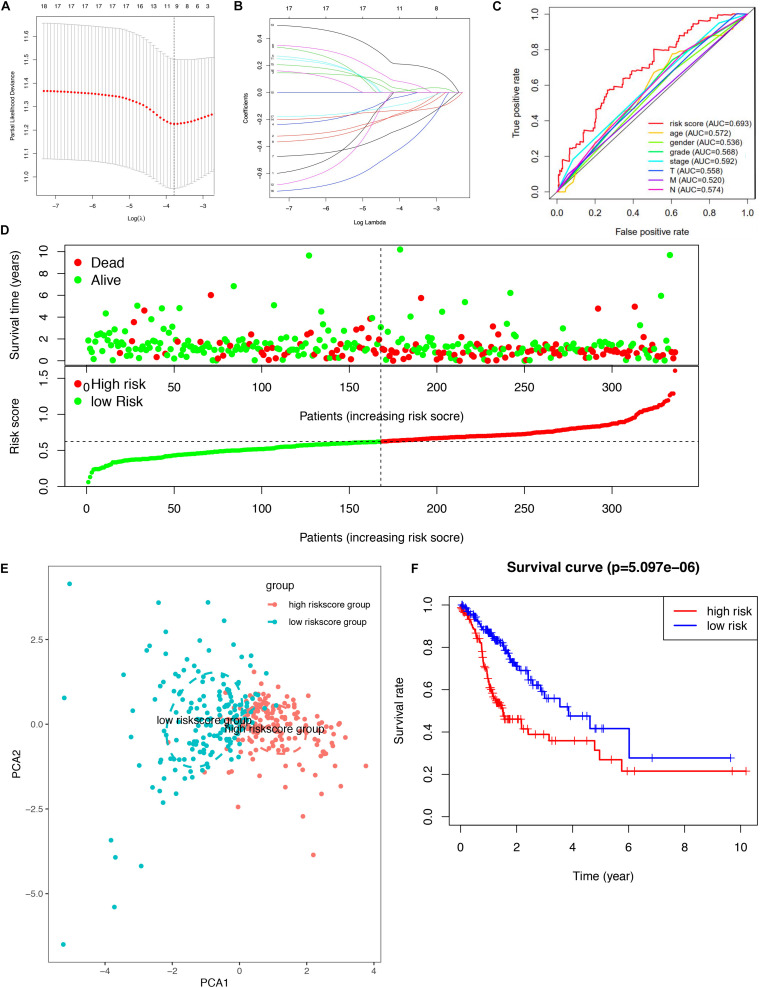
Lasso regression identified the prognostic model in STAD. **(A,B)** Lasso regression complexity was controlled by lambda using the glmnet R package. **(C)** The multi-ROC of risk score and clinical features. **(D)** Risk plot between the high- and low-risk score groups. **(E)** Principal component analysis for the risk scores. **(F)** Overall survival analysis between high and low-risk score groups.

A total of 18 survival-related DEGs (risk ratio > 1.4 or risk ratio < 0.7, *p* < 0.05) were selected to perform Lasso regression, including MAPK10, CPZ, ARHGAP20, FAT3, PLCL1, CCL14, BMPER, NUDT10, HSPB2, JAKMIP2, PRR5-ARHGAP8, DNASE1L2, ZGRF1, DNAAF3, CCDC15, ARHGEF38-IT1, SYCE2, and PASK. Furthermore, the nine mRNAsi survival-related DEG prognostic model (ARHGEF38-IT1, CCDC15, CPZ, DNAAF3, DNASE1L2, NUDT10, PASK, PRR5-ARHGAP8, and SYCE2) was constructed with Lasso regression to improve the predicted accuracy for overall survival in STAD, when log (lambda) was between -3 and -4 (Figures 4A,B). The ROC curve showed that the AUC value was higher than other clinical indices (age, sex, grade, stage, T stage, N stage, and M stage), which indicated that the nine mRNAsi survival-related DEG prognostic model was better than other clinical indices currently used to evaluate the prognostic status of STAD patients (Figure 4C). A survival risk heatmap showed that STAD patients with higher risk scores suffered from higher mortality (Figure 4D). The PCA plot also proved that the nine mRNAsi survival-related DEG prognostic model had the power to distinguish two separate groups of STAD patients (Figure 4E). Additionally, overall survival showed statistical significance between the high- and low-risk score groups using the Kaplan–Meier survival curve (Figure 4F and Supplementary Table 8).

**FIGURE 5:**
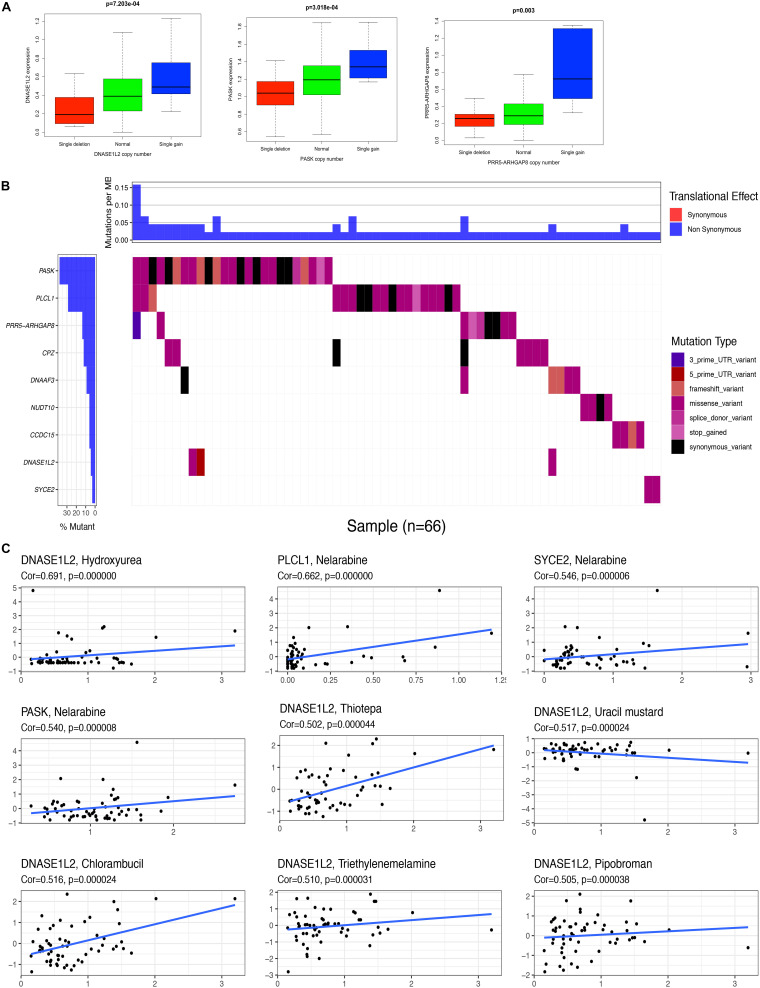
Correlation analysis between the expression of genes in prognostic signatures and CNV, mutation, and drug sensitivity. **(A)** Correlation analysis between the expression of genes (DNASE1L2, PASK, and PRR5-ARHGAP8) in prognostic signatures and CNV. **(B)** The mutation distribution of genes (ARHGEF38-IT1, CCDC15, CPZ, DNASE1L2, NUDT10, PASK, PLCL1, PRR5-ARHGAP8, and SYCE2) in prognostic signatures. **(C)** Correlation analysis between the expression of genes (DNASE1L2, PLCL1, SYCE2, and PASK) in prognostic signatures and drug sensitivity.

Correlation analysis between the expression of DEGs in the prognostic model and CNV showed four CNV-driven DEGs, including DNASE1L2, PASK, and PRR5-ARHGAP8. For example, the expression of DNASE1L2 was downregulated in the single deletion CNV group compared with that in the normal CNV group. However, it was upregulated in the single-gain CNV group in STAD (*p* < 0.05). The other two DEGs in the prognostic model showed a similar trend (Figure 5A and Supplementary Table 9). The results suggested that some dysregulation of key genes might be driven by CNV in STAD. The gene mutation status was also shown with a waterfall plot. The overall average mutation frequency of DEGs in the prognostic model ranged from 0.10 to 38%. The PSK mutation frequency is listed at the top, and the PLCL1 mutation frequency was more than 20% in STAD (Figure 5B and Supplementary Table 10). The results suggested that dysregulation of key genes might be driven by mutations in STAD. The correlation analysis between DEG expression in the prognostic model showed that some genes were significantly associated with drug sensitivity. For example, there was a significant correlation between the expression of DNASE1L2 and hydroxyurea, uracil mustard, chlorambucil, triethylenemelamine, pipobroman, thiotepa, and chelerythrine. Additionally, there was a significant correlation between the expression of PLCL1, SYCE2, PASK, and nelarabine (Figure 5C and Supplementary Table 11). These results provide potential directions for the development of drugs in the future.

### Association of Risk Score With Clinical Features, Immune Cells, and TMB

**FIGURE 6:**
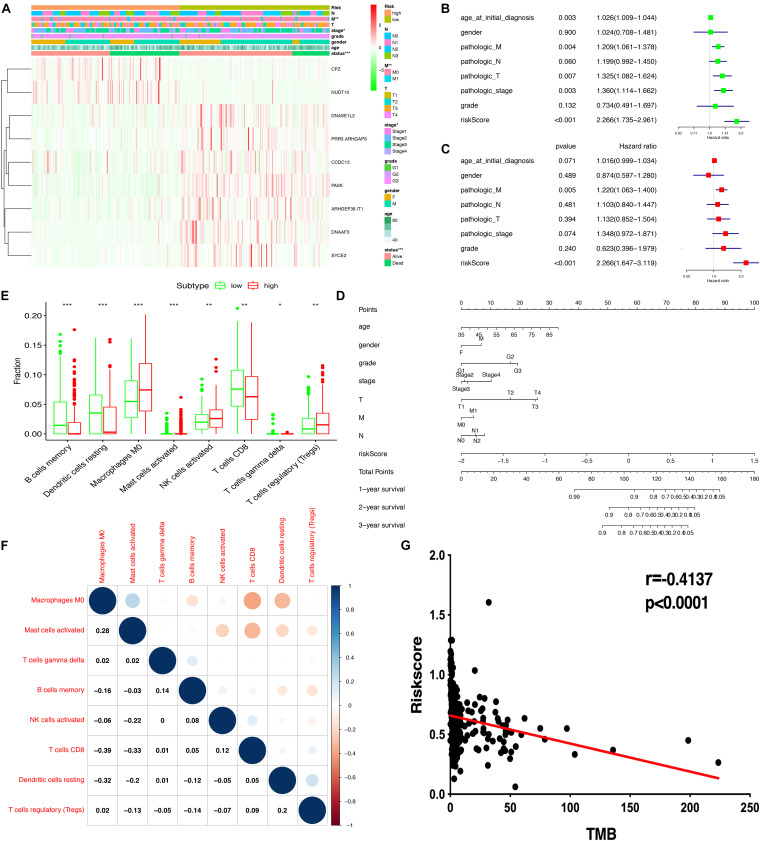
Correlation of risk scores based on the prognostic model with clinical characteristics, immune cells, and TMB. **(A)** Heatmap of clinical correlation between high and low-risk score groups. **(B)** Univariate Cox regression analysis of risk factors. **(C)** Multivariate Cox regression analysis of risk factors. **(D)** The risk score and clinical information assessment nomogram to evaluate STAD prognosis (1-, 2-, and 3-year survival rates). **(E)** Boxplot shows the ratio differences of eight immune cells between the high- and low-risk score groups. The Wilcoxon rank sum was used for the significance test. **(F)** The correlation between these eight immune cells. **(G)** The correlation of risk scores based on the prognostic model with TMB. ^∗^*p* < 0.05, ^∗∗^*p* < 0.01, and ^∗∗∗^*p* < 0.001.

The heatmap shows that risk groups were significantly associated with clinical features, including pathologic M stage and cancer status (Figure 6A and Supplementary Table 12). Univariate Cox regression analysis revealed that age at initial diagnosis, pathologic M stage, pathologic T stage, pathologic stage, and risk score were significantly correlated with overall survival (Figure 6B). This indicated that the risk score could be a risk factor for STAD. Multivariate Cox regression analysis revealed that pathologic M stage and risk score were significantly correlated with overall survival (Figure 6C). This indicated that the risk score could be an independent risk factor for STAD. Furthermore, the nomogram plot shows points for basic clinical characteristics (age at initial diagnosis, sex, pathologic M stage, pathologic T stage, pathologic N stage, and pathologic stage) and risk score to estimate the patient survival rate according to the combined scoring system (Figure 6D). It is a convenient method to guide clinical applications. The proportion of immune cells in STAD was significantly different between the high- and low-risk groups, including Tregs, T cell gamma delta, CD8^+^ T cells, resting dendritic cells, activated NK cells, M0 macrophages, memory B cells, and activated mast cells (Figure 6E and Supplementary Table 13). Different proportions of immune cells between risk score subtypes are correlated with each other; for example, CD8^+^ T cells and M0 macrophages, CD8^+^ T cells and activated mast cells, resting dendritic cells, and M0 macrophages (Figure 6F). The risk score was negatively correlated with the TMB score (Figure 6G and Supplementary Table 13). This indicated that the high-risk score group might have a poor prognosis with a low TMB score.

### GSEA Identified Some Significant Gene Sets Between the High- and Low-Risk Score Groups

**FIGURE 7:**
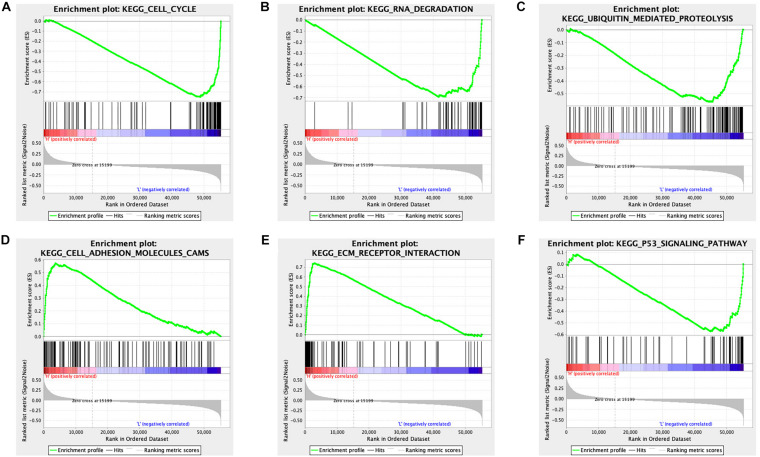
GSEA identified different gene sets between the high and low-risk score groups in STAD. **(A)** The cell cycle pathway was significantly different between the high- and low-risk score groups. **(B)** The RNA degradation pathway was significantly different between the high- and low-risk score groups**. (C)** The ubiquitin-mediated proteolysis pathway was significantly different between the high- and low-risk score groups. **(D)** The cell adhesion molecules pathway was significantly different between the high- and low-risk score groups. **(E)** The EMC receptor interaction pathway was significantly different between the high- and low-risk score groups. **(F)** The p53 signaling pathway was significantly different between the high- and low-risk score groups.

The STAD samples were divided into two groups according to the risk score. Some significant gene sets were enriched in the high-risk score group, including gap junction, arachidonic acid metabolism, neuroactive ligand receptor interaction, cell adhesion molecule cams, calcium signaling pathway, focal adhesion, complement and coagulation cascades, and EMC receptor interaction. Other significant gene sets were enriched in the low-risk score group, including mismatch repair, DNA replication, homologous recombination, spliceosome, cell cycle, proteasome, protein export, RNA degradation, p53 signaling pathway, and ubiquitin-mediated proteolysis (Supplementary Table 13). Example plots are shown in Figure 7.

## Discussion

The intrinsic characteristics of CSCs include self-renewal and multipotent properties, as well as proliferative potential, which give certain cellular subpopulations the ability to initiate, develop, and progress cancer (Huang et al., 2020). Dormant CSCs may be activated by a series of gene mutation accumulations and undergo a significant number of DNA sequence alterations (Sengupta and Cancelas, 2010). These progressively accumulated mutations drive immune escape and drug resistance in CSCs (Rosa et al., 2016). CSCs have been reported in most human tumors using fluorescence-activated cell sorting (FACS) and sphere-forming assays (Jariyal et al., 2019). Common CSC identification markers include ALDH1A1, CD34, CD24, CD44, CD123, CD133, CD117, and EpCAM (Kim et al., 2016). These specific CSC markers can be selectively targeted and used to treat aggressive, metastatic, and relapse tumors. For example, targeting the overexpressed CD123 marker on CD34^+^ CD38^–^ leukemic stem cells in acute myelogenous leukemia impairs leukemic stem cells homing to the bone marrow and induces a decrease in the overall AML cell repopulation (Jin et al., 2009). Packaged nanoparticles with miR-34a can downregulate the level of CD44 marker of CSCs in a mouse model of prostate cancer (Wang et al., 2016). Although the success of targeting CSCs has been limited, this may soon be a viable option for intervention strategies, disease identification, metastasis prevention, and identification of selective drug targets (Kabakov et al., 2020). Recently, many CSCs have demonstrated phenotypic plasticity and altered their transcriptomes under therapeutic challenges to escape destruction, cooperating with intratumoral heterogeneity and the immune microenvironment (Miranda et al., 2019). Bioinformatics analysis designed to reveal the association between cancer cell stemness, gene mutation, and the immune microenvironment in STAD provided a prognostic model, potential mechanisms, and the associated factors (immune microenvironment and mutation status) of targeting CSCs for future research.

To our knowledge, this is the first systematic analysis of the association between cancer cell stemness, gene mutation, and the immune microenvironment in STAD. These factors were intimately linked to STAD. A total of 6,739 DEGs were identified between normal and tumor tissues in STAD, and further WGCNA identified that black and brown gene co-expression modules were most significantly related to mRNAsi and EREG-mRNAsi. Some of the identified 178 survival and mRNAsi-related hub DEGs in this study have already attracted great attention in the field of CSCs. For example, the KIT gene encodes the human homolog of the proto-oncogenic receptor tyrosine kinase c-kit. The C-kit was identified as a transmembrane receptor for mast cell growth factor (stem cell factor). Dysregulation or mutation of KIT is known to be associated with gastrointestinal tumors (Hirota et al., 1998). The c-kit receptor, as a stem cell factor, mediates the development of some tumors by regulating survival and *de novo* proliferation of mast cells to affect various biological processes, such as innate and adaptive immune responses, expression of surface receptors (chemokines, high-affinity IgE receptor, immunoglobulins, and cytokines), and angiogenesis. As an anticancer strategy, targeting c-kit may play an important role in human clinical trials (Ammendola et al., 2016). The transcription factor SOX-9 could construct a DNA-binding complex with HMG-box class proteins to recognize the sequence CCTTGAG. Almost all gastric carcinoma cells overexpressed the stem cell marker SOX9 (Sashikawa Kimura et al., 2011). *Helicobacter pylori* infection is well known to be a major risk factor for STAD. In a previous study, SOX9 was significantly upregulated in specimens infected with *H. pylori* to increase levels of β-catenin, induce gastric cancer cell proliferation, and enhance stem cell-like properties (Santos et al., 2016). TFF2 protein is a stable secretory protein expressed in the gastrointestinal mucosa, which can affect the healing of the epithelium, protect the mucosa from insults, and stabilize the mucus layer, inhibiting gastric acid secretion (Qiao and Gumucio, 2011). A transgenic mouse with the TFF2 promoter was generated to check whether it was a marker of gastric progenitor cells; the results suggested that TFF2 transcript-expressing cells were progenitors for parietal, mucus neck, and zymogenic cells (Quante et al., 2010). Some of the identified survival and mRNAsi-related hub DEGs in this study closely interacted with stem cell markers. For example, SLC7A11, a glutamate-cystine transporter, is a member of a heteromeric, sodium-independent, anionic amino acid transport system. SLC7A11 combined with CD44 (cancer cell stem marker) controls the reduced glutathione and defense against reactive oxygen species in a transgenic mouse model of gastric cancer (Ishimoto et al., 2011). These examples indicate that findings in our study were consistent with those of previous studies. Interestingly, we have also reported new findings that provide a foundation for future studies on CSCs in STAD.

The identified 178 survival and mRNAsi-related hub DEGs in this study were significantly enriched in some crucial CSC-related pathways. Various anticancer drugs targeting CSCs are closely related to cell cycle checkpoints and pathways (Yuan et al., 2015). For example, the effects of metformin have been evaluated by two- and three-dimensional cell culture systems to observe the development of tumorspheres in gastric cancer cell lines. The antitumor effect of metformin on STAD could target gastric CSCs by inducing cell cycle arrest (Courtois et al., 2017). The effects of all-trans-retinoic acid on CSCs in STAD were also evaluated using conventional two- and three-dimensional cell culture systems. All-trans-retinoic acid can inhibit tumor sphere initiation through cell cycle arrest via an increase in cyclin-dependent kinase (CDK) inhibitors and decrease in cell-cycle progression activators (Nguyen et al., 2016). The Notch pathway regulates gastric stem cell proliferation in mouse genetic models through HES1, NICD, and mTORC1 target ribosomal proteins. Additionally, Notch pathway components, including downstream target genes, Notch ligands, and receptors have been reported to be oncogenes in various tumors (Hibdon et al., 2019). In this study, survival and mRNAsi-related hub DEGs were significantly related to the Notch pathway, including AGO2, CNTN1, DTX4, LFNG, TACC3, and TFDP1. Some of the components of the Notch pathway have been proposed to be crucial for CSCs in other studies. For example, LFNG encodes evolutionarily conserved glycosyltransferases that define boundaries during embryonic development in the Notch signaling pathway. Musashi2 contributes to the maintenance of CD4^+^ 4^*v*6+^ liver CSCs via the Notch signaling pathway, which increases proliferation, migration and invasion, self-renewal, and resistance to sorafenib. Mechanically, Musashi2 interacts with the mRNA and proteins of LFNG to activate Notch1. This suggests that LFNG could be a potential target for stem cell-targeted therapy for cancers (Wang X. et al., 2019). TACC3, a motor spindle protein, which may be necessary for stabilization of the mitotic spindle, has been proven to be important in the differentiation of various cancer cells. Moreover, TACC3 is commonly overexpressed in cancers and positively correlated with poor overall survival and disease-free survival (Thakur et al., 2013). TACC3 inhibitor could reduce sphere formation, clonogenicity, cell growth and proliferation, and cancer stem cell-like phenotype by suppressing the Wnt/β-catenin and PI3K/AKT signaling pathways (Zhou et al., 2015). The downstream target gene of the Notch signaling pathway, TFDP1 (a heterodimeric partner of the E2F family) can be directly regulated by microRNA-4711-5p to induce G1 arrest; suppress cell migration and invasion abilities, sphere formation, proliferation, and reactive oxygen species activity; and decrease the expression of stem cell markers (Morimoto et al., 2020). Several miRNAs have potent tumor-suppressive effects. Additionally, miRNAs are short RNAs that are easily enveloped by nanoparticles to target cancer-related pathways and genes (Rezaie et al., 2018). The identified survival and mRNAsi-related hub DEGs were also significantly related to the homology directed repair (HDR) pathway. Double-stranded DNA lesions can be repaired to maintain genomic stability by the HDR mechanism, which is important for suppressing cancer formation and development. For example, if the HDR pathway is dysfunctional, expression of a defective receptor on the cell surface might ignore signals to stop dividing and continue to form a tumor (Mladenov et al., 2016). Poly (ADP-ribose) polymerase (PARP) inhibitors have been developed as promising cancer therapeutics, especially for HDR-deficient tumors (Bian et al., 2018). CSCs increase the ability to repair DNA damage through the upregulation of DNA damage responses, such as HDR, and enhancement of the capacity to arrest at specific cell cycle checkpoints. PARP family members are also involved in cancer stem cell biology. For example, the combination of a PARP inhibitor (Niraparib) and the cyclin-dependent kinase inhibitor dinaciclib downregulates MYC-driven homologous recombination and reduces cancer stem cell-like phenotypes. Targeting HDR in CSCs may be a promising cancer strategy (Zeniou et al., 2019).

Furthermore, the nine-gene signature prognostic model (ARHGEF38-IT1, CCDC15, CPZ, DNASE1L2, NUDT10, PASK, PLCL1, PRR5-ARHGAP8, and SYCE2) constructed in this study was significantly related to the overall survival, clinical characteristics, tumor microenvironment immune cells, TMB, and cancer-related pathways. Some genes in the prognostic model were driven by CNV or gene mutation and were associated with drug sensitivity. The development of cancers is affected by multiple factors and various genes. It is difficult to completely explain the pathogenic course, clinical features, and prognosis of cancer using a single-gene model. A multigene-signature prognostic model would provide an optimized integrated risk score using a regression method. Our nine-gene signature prognostic model was significantly associated with STAD survival and well-distributed samples into two separate subtypes, as tested by PCA. ROC indicated a high sensitivity and specificity for the risk score as a prognostic factor, even better than the common clinical features, including age, sex, grade, stage, T stage, N stage, and M stage. As one of the independent risk factors, the nomogram plot also proved that it is a fine-to-important risk score as one of the clinical variables in clinical practice. Therefore, there was an important significance to the prediction of prognosis in STAD combined detection of the risk score based on mRNAsi and clinical features. However, the power of the model could be improved, if one could collect clinical samples to verified the prognostic model. Further study was necessary to verified the prognostic model in a large register-based series. In terms of mechanisms, DNASE1L2, PASK, and PRR5-ARHGAP8 might be driven by CNVs. The mutation frequency of PASK was more than 30% in STAD. A recent study showed that PASK, a downstream phosphorylation target of mTORC1, stimulates the differentiation of muscle stem cells by activating the myogenin promoter and driving stem cell self-renewal (Kikani et al., 2019). The correlation between the expression of genes in the prognostic model and drug sensitivity provides more findings for some drugs that are currently undergoing clinical trials. For example, 26 women with metastatic breast cancer who developed resistance to low-dose combination chemotherapy were enrolled to receive a high-dose hydroxyurea (18 mg/mL), cyclophosphamide (6 mg/mL), and thiotepa (600 mg/mL) combination. Phase I and II trials have demonstrated that 1 and 2 years progression-free survival was improved (Vaughan et al., 1994). In another study, 26 patients with lymphoblastic leukemia and 13 patients with lymphoblastic lymphoma were enrolled and treated with nelarabine. Nelarabine has significant antitumor activity and is well tolerated in relapsed or refractory lymphoblastic leukemia and lymphoblastic lymphoma (DeAngelo et al., 2007). Other drugs, such as uracil mustard, chlorambucil, triethylenemelamine, pipobroman, thiotepa, and chelerythrine, have also been studied in STAD.

In terms of the immune microenvironment, some immune cell proportions were significantly different between the high- and low-risk score groups, such as Tregs, T cell gamma delta, CD8^+^ T cells, resting dendritic cells, activated NK cells, M0 macrophages, memory B cells, and activated mast cells. Cross-talk between immune cells and CSCs is obvious in various cancers. Recent studies have revealed that CSC-targeted immunotherapy based on various immunological effector cells is a possible and promising method for patients with cancer (Saijo et al., 2013). For example, CD133 is upregulated in the population of CSCs and is a potential target for cytotoxic T cells. Specifically, in lysed CD133^+^ CSCs, natural processing, antigen presentation, and the ability of cytotoxic T cells to kill CSCs bearing the antigen are effective (Ji et al., 2014). Some cytokines also regulate proliferation, tumorigenicity, and migration of mesenchymal CSCs *in vivo* and *in vitro*. Additionally, immune infiltration varies widely among CSC molecular subtypes in a TGF-beta-dependent manner (Beier et al., 2012). NK cells, CD8^+^ T cells, γδ T cells, and antibodies have been shown to target CSCs and kill target cells using cytotoxic granules (granzymes and perforin). Therefore, treatment-resistant CSCs are susceptible to CSC-targeted immunotherapy (Hirohashi et al., 2010). Patients with high tumor mutational burden have had favorable prognoses, especially in those treated with immune checkpoint inhibitors. Patients with advanced gastric cancer with a high tumor mutational burden were correlated with enhanced progression-free survival and objective response rate when receiving immunotherapy (Wang F. et al., 2019). The negative correlation between risk score and TMB demonstrated in our study was consistent with previous reports, which indicated cross-talk between gene mutations and stemness in STAD. Furthermore, the cancer-related gene sets between the high- and low-risk score groups widely involved cellular processes, including the levels of RNA, DNA, and protein. These results provide insights into the alterations in CSC-related mechanisms in STAD.

## Conclusion

We focused on alterations in stemness-related genes in STAD, using the mRNAsi index. The prognostic models for STAD could be useful for further investigation of clinical applications in stomach cancers. The association between CSCs, gene mutations, and the immune microenvironment in STAD provides an improved understanding of different drug targets and can enable the development of CSC-targeted therapy.

## Data Availability Statement

The original contributions presented in the study are included in the article/Supplementary Material, further inquiries can be directed to the corresponding author.

## Author Contributions

ZY designed the project, analyzed the data, prepared the figures and tables, and wrote the manuscript. LC conceived the research, supervised the results, critically revised, wrote the manuscript, and was responsible for its financial support and the corresponding works. All others conceived the research, critically revised the manuscript, and approved the final manuscript.

## Conflict of Interest

The authors declare that the research was conducted in the absence of any commercial or financial relationships that could be construed as a potential conflict of interest.

## Footnotes

1
https://portal.gdc.cancer.gov/

2
https://xenabrowser.net/datapages/

3
https://www.bioconductor.org/packages/release/bioc/html/maftools.html

4
https://www.bioconductor.org/packages/release/bioc/html/limma.html

5
https://horvath.genetics.ucla.edu/html/CoexpressionNetwork/Rpackages/WGCNA/

6
https://www.rdocumentation.org/packages/survival/versions/3.2-3

7
http://ci.smu.edu.cn/genclip3/analysis.php

8
https://string-db.org/cgi/input?sessionId=bEj4yF0DUhDV&input_page_show_search=on

9
https://cran.r-project.org/web/packages/glmnet/index.html

10
https://www.rdocumentation.org/packages/pROC/versions/1.16.2/topics/roc

11
https://cran.r-project.org/web/packages/corrplot/vignettes/corrplot-intro.html

12
https://discover.nci.nih.gov/cellminer/

13
http://cibersort.stanford.edu/

14
https://www.rdocumentation.org/packages/pheatmap/versions/1.0.12/topics/pheatmap

15
http://rstudio-pubs-static.s3.amazonaws.com/5896_8f0fed2ccbbd42489276e554a05af87e.html

16
https://cran.r-project.org/web/packages/rms/index.html

17
https://cran.r-project.org/web/packages/ggpubr/index.html
