# Corrigendum: Bioinformatics Analysis Reveals an Association Between Cancer Cell Stemness, Gene Mutations, and the Immune Microenvironment in Stomach Adenocarcinoma

**DOI:** 10.3389/fgene.2022.828664

**Published:** 2022-02-14

**Authors:** Zaisheng Ye, Miao Zheng, Yi Zeng, Shenghong Wei, Yi Wang, Zhitao Lin, Chen Shu, Yunqing Xie, Qiuhong Zheng, Luchuan Chen

**Affiliations:** ^1^ Department of Gastrointestinal Surgical Oncology, Fujian Cancer Hospital and Fujian Medical University Cancer Hospital, Fuzhou, China; ^2^ Department of Clinical Laboratory, Fujian Maternity and Child Health Hospital, Affiliated Hospital of Fujian Medical University, Fuzhou, China; ^3^ Department of Fujian Provincial Key Laboratory of Tumor Biotherapy, Fujian Cancer Hospital and Fujian Medical University Cancer Hospital, Fuzhou, China

**Keywords:** stomach adenocarcinoma, cancer stemness, clinical characteristics, tumor microenvironment, tumor mutation burden

In the original article, there was a mistake in Figure 1 and caption ***/*** Supplementary Table S1 as published. The whole process of analysis was based on the raw mRNA expression-based stemness index (mRNAsi), but the survival curve was based on the corrected mRNAsi (mRNAsi/tumor purity). The inconsistent data was confusing. According to consistency principle, we decided to use the raw mRNAsi data to construct survival curve in STAD (Figure 1B; Supplementary Table S1). Second, the limma package was used for the analysis of DEGs between normal and tumor tissues in STAD as published in the section of *“Identification of DEGs Between Normal and Tumor Tissues in STAD,”* but we described as “*between the high and low mRNAsi groups*” (Figure 1A).

**FIGURE 1:**
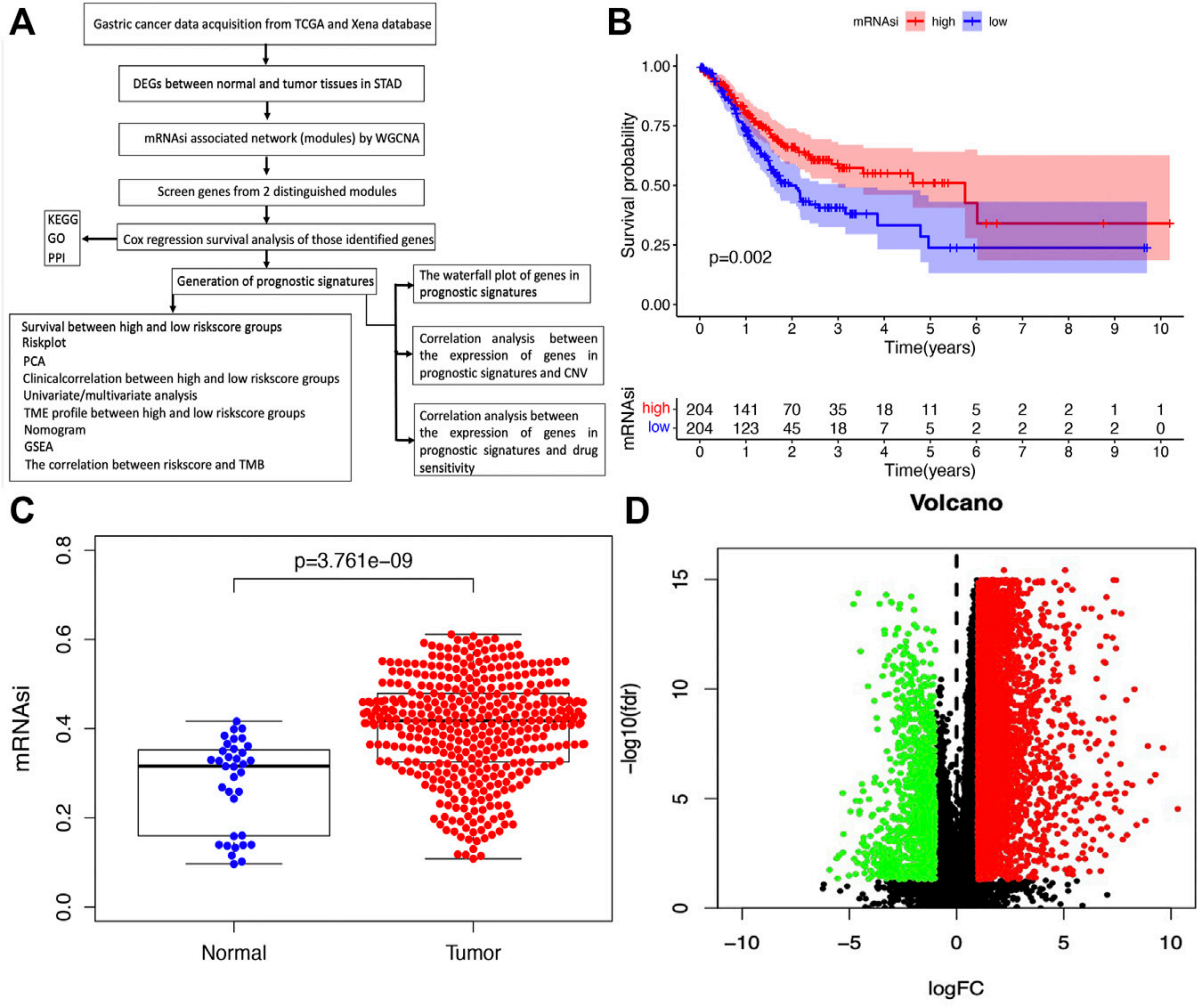
Identification of DEGs between normal and tumor tissues in STAD. **(A)** The flow chart for identification of mRNAsi related signature in STAD. **(B)** Kaplan–Meier curves show that the low mRNAsi group had greater mortality than the high mRNAsi group. **(C)** Differences in mRNAsi between normal and tumor tissues in STAD. **(D)** Identification of DEGs between normal and tumor tissues in STAD; green and red indicate downregulated and upregulated genes, respectively.

The corrected Figure 1 and caption appear below.

The corrected **Supplementary Table S1** available in Supplementary Material.

In addition, errors were published in the **Abstract** and **Results** section of the original article.

A correction has been made to “**Abstract**” given below.

“Cancer stem cells (CSCs), characterized by infinite proliferation and self-renewal, greatly challenge tumor therapy. Research into their plasticity, dynamic instability, and immune microenvironment interactions may help overcome this obstacle. Data on the stemness indices (mRNAsi), gene mutations, copy number variations (CNV), tumor mutation burden (TMB), and corresponding clinical characteristics were obtained from The Cancer Genome Atlas (TCGA) and UCSC Xena Browser. The infiltrating immune cells in stomach adenocarcinoma (STAD) tissues were predicted using the CIBERSORT method. Differentially expressed genes (DEGs) between the normal and tumor tissues were used to construct prognostic models with weighted gene co-expression network analysis (WGCNA) and Lasso regression. The association between cancer stemness, gene mutations, and immune responses was evaluated in STAD. A total of 6,739 DEGs were identified between the normal and tumor tissues. DEGs in the brown (containing 19 genes) and blue (containing 209 genes) co-expression modules were used to perform survival analysis based on Cox regression. A nine-gene signature prognostic model (ARHGEF38-IT1, CCDC15, CPZ, DNASE1L2, NUDT10, PASK, PLCL1, PRR5-ARHGAP8, and SYCE2) was constructed from 178 survival-related DEGs that were significantly related to overall survival, clinical characteristics, tumor microenvironment immune cells, TMB, and cancer-related pathways in STAD. Gene correlation was significant across the prognostic model, CNVs, and drug sensitivity. Our findings provide a prognostic model and highlight potential mechanisms and associated factors (immune microenvironment and mutation status) useful for targeting CSCs.”

A correction has been made to the section of **Results**, “*mRNAsi Was Significantly Associated With STAD*,” “*Paragraph Number 1 and 4*” given below.

## Results

### mRNAsi Was Significantly Associated With STAD

“The flowchart shown in Figure 1A summarizes the overall bioinformatics analysis of the association between cancer stemness, gene mutations, and immune response in STAD. The data of mRNAsi were used to perform the overall survival analysis in STAD (Supplementary Table S1). The mRNAsi subgroups were clustered according to the median value of mRNAsi in STAD. The overall survival was significantly different between the high and low mRNAsi groups (Figure 1B, *p* = .002). A significant difference in mRNAsi was observed between normal and tumor tissues in STAD (Figure 1C). A total of 6,739 DEGs were identified between normal and tumor tissues in STAD, of which 1,146 were upregulated and 5,593 were downregulated (Figure 1D; Supplementary Table S2).”

The authors apologize for these errors and state that this does not change the scientific conclusions of the article in any way. The original article has been updated.

## Supplementary Material

Supplementary Table 1 The mRNA expression-based stemness index (mRNAsi) of stomach adenocarcinoma (STAD) and corresponding survival data from TCGA database.

